# Detection of *ALK* Gene Rearrangement in Cell-free RNA from Lung Cancer Malignant Pleural Effusion

**DOI:** 10.1155/2020/6124106

**Published:** 2020-01-21

**Authors:** Mingliang Chu, Yanqiu Zhu, Jianjun Hu, Zhuxue Zhang, Meng Luo, Xiaobo Ma

**Affiliations:** ^1^Department of Clinical Laboratory, First Affiliated Hospital of Guizhou University of Traditional Chinese Medicine, Guiyang 550001, China; ^2^Department of Pathology, Guizhou Provincial People's Hospital, Guiyang 550002, China; ^3^Department of Thoracic Surgery, Guizhou Provincial People's Hospital, Guiyang 550002, China; ^4^Department of Surgery-Transplant, The University of North Carolina at Chapel Hill, Chapel Hill, NC 27599, USA

## Abstract

The aim of this study was to evaluate the feasibility of measuring *ALK* gene rearrangement in cell-free RNA (cf-RNA) of the supernatant from malignant pleural effusion (MPE). Supernatant, cell blocks, and matched sera samples were collected. Cf-RNA was isolated from the supernatant and sera, and cellular RNA was isolated from cell blocks. The *ALK* gene rearrangement in the cf-RNA was tested by the real-time polymerase chain reaction. Results showed that the concentration of cf-RNA was higher in the supernatant than in matched sera. *ALK* status concordance rates were 100% between the supernatant and cell blocks, while they were 0% between sera and cell blocks in *ALK* gene rearrangement cases. This suggests that using cf-RNA in MPE supernatant, but not in sera, could offer a reliable and robust surrogate strategy for the detection of *ALK* gene rearrangement.

## 1. Introduction

Lung cancer is the leading cause of cancer death among both men and women, of which non-small-cell lung cancer (NSCLC) represents approximately 85% of the total cases diagnosed [1]. Driver gene abnormality tests are the premise of targeted therapy for advanced NSCLC, particularly in the adenocarcinoma subtype [2]. Among these, *EGFR* gene mutations and *ALK* gene rearrangement are widely recognized alterations that respond to target agents in NSCLC [3]. Malignant pleural effusion (MPE) is a common manifestation in patient with advanced lung cancer. Most MPE contain tumor cells, which can be used for *EGFR* and *ALK* gene status tests [4–7].

Cell-free nucleic acids are extracellular nucleic acids found in cell-free plasma/serum and other biological fluids [8]. Detection of cell-free nucleic acids (DNA or RNA) using quantitative real-time PCR or quantitative real-time RT-PCR (qRT-PCR) methods have been suggested as a promising diagnostic tool for cancer detection [9]. A number of studies have reported that *EGFR* mutations can be detected in cell-free DNA (cf-DNA) of malignant pleural effusion rearrangement samples from lung cancer patients [10, 11]. Though researchers have showed that there was cell-free RNA (cf-RNA) in MPE [12], it is still not known whether *ALK* gene rearrangements can be detected by qRT-PCR using cf-RNA. Therefore, the subject of the present study was to explore the use of the newly developed method for the detection of *ALK* gene rearrangement in MPE.

## 2. Materials and Methods

### 2.1. Patients and Samples

A total of 168 MPE samples were collected and centrifuged. The tumor cell content in cell blocks was sufficient to perform the subsequent immunohistochemistry (IHC) and *ALK* gene rearrangement test. Twenty-three cases of cell-free supernatant and sera (matched with 12 *ALK* positive rearrangement and 11 *ALK* negative rearrangement cell blocks) were collected. The diagnosis of NSCLC was based on cytology via a combination of IHC staining results and clinical information. All of the samples were obtained from Guizhou Provincial People's Hospital during November 2015–June 2019. The age at the time of initial diagnosis and smoking status were obtained from the hospital medical records. The study was approval by the Ethics Committees of Guizhou Provincial People's Hospital.

### 2.2. RNA Extraction and *ALK* Gene Rearrangement Detection

The cell blocks were embedded by paraffin, and then cellular RNA was extracted by the use of an RNA FFPE Tissue Kit (AmoyDx, Xiamen, China) according to the manufacturer's protocols. The supernatant and sera were firstly centrifuged for 10 min at full speed (8000 g/min), and then cf-RNA was extracted by the use of a Circulate Nucleic Acids Kit (AmoyDx, Xiamen, China) according to the manufacturer's protocols. The quantity of isolated RNA was assessed by using a NanoDrop2000 spectrophotometer (Thermo, L.A., USA). The *ALK* gene rearrangement was detected by qRT-PCR using an *ALK* gene rearrangement Detection Kit (AmoyDx, Xiamen, China). The PCR reaction was performed on an Agilent Mx3000P QPCR instrument (Agilent Technologies, Santa Clara, CA). The following PCR procedure was used: an initial reverse transcription at 42°C for 5 min, denaturation at 95°C for 5 min, and then 10 annealing cycles at 95°C for 25 seconds, 64°C for 20 seconds, and 72°C for 20 seconds, followed by 36 extension cycles at 93°C for 25 seconds, 60°C for 35 seconds, and 72°C for 20 seconds to perform the data collection. The quantitative judgment was according to the gene rearrangement fluorescence signal. Assay reactions achieving threshold cycle (Ct) values of ≤35 cycles were considered as positive, otherwise as negative. Amplification of the control gene (beta-actin) in the assay demonstrates the presence of RNA.

### 2.3. Statistical Analysis

Data were analyzed using SPSS 13.0 software (SPSS Inc., Chicago, IL, USA). The chi-square test, Friedman test, and paired Student's *t*-test were performed to analyze variables, where appropriate. In addition, Kappa statistic and McNemar's test were performed to determine consistency between the supernatant, sera, and cell blocks. *p* < 0.05 was considered statistically significant.

## 3. Results

### 3.1. Patient Features

In total, 168 cases with MPE were eligible for analysis. The *ALK* gene rearrangement status was assessed by analysis of RNA extracted from cell blocks using the qRT-PCR method. The clinicopathological features of the patients are shown in Table 1. The current *ALK* positive rate was 7.1% (12/168) in patients with MPE. Further analysis found that the positive rate was significantly higher in patients with age under 60 years (14.1%) than in patients with age over 60 years (2.4%) (*p*=0.012; Table 1).

### 3.2. Cf-RNA in Supernatant and Sera

**Figure 1:**
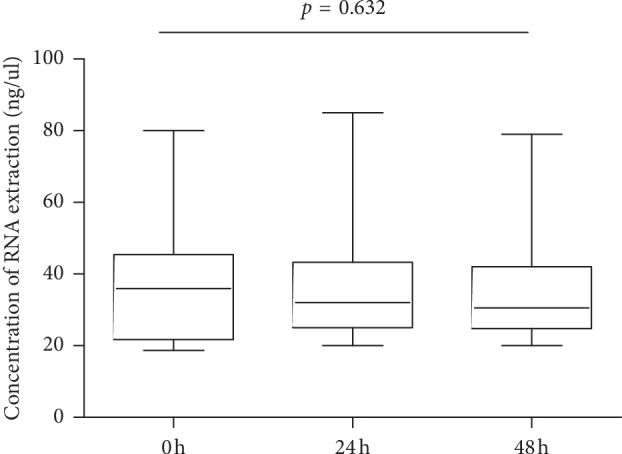
Concentration of cf-RNA extraction (ng/ul) in the MPE supernatant as determined by using a spectrometer is plotted on the *y*-axis. The processing time points are shown on the *x*-axis (0, 24, and 48 hours). Statistically significant differences were determined by using the Friedman test.

**Figure 2:**
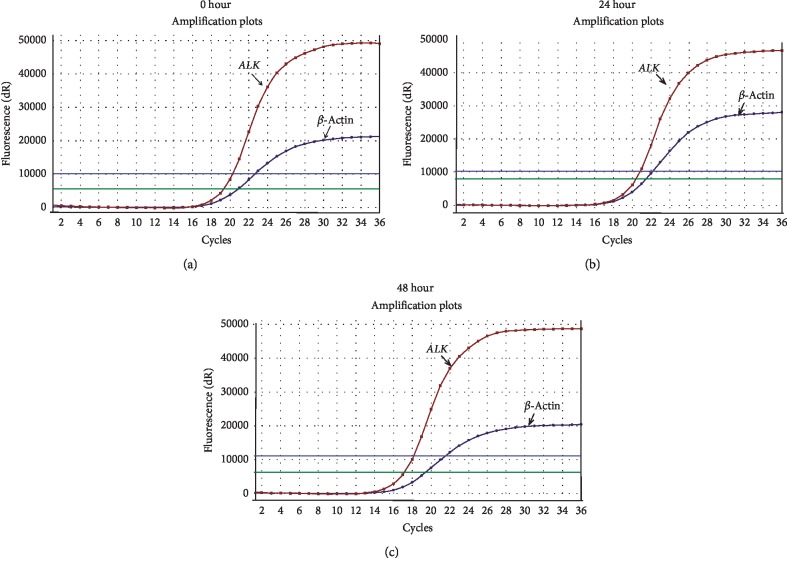
Detection of *ALK* gene rearrangement at three processing time points in cf-RNA of the MPE supernatant. (a) Amplification curve of *ALK* rearrangement at 0 hour. (b) Amplification curve of *ALK* rearrangement at 24 hours. (c) Amplification curve of *ALK* rearrangement at 48 hours.

**Figure 3:**
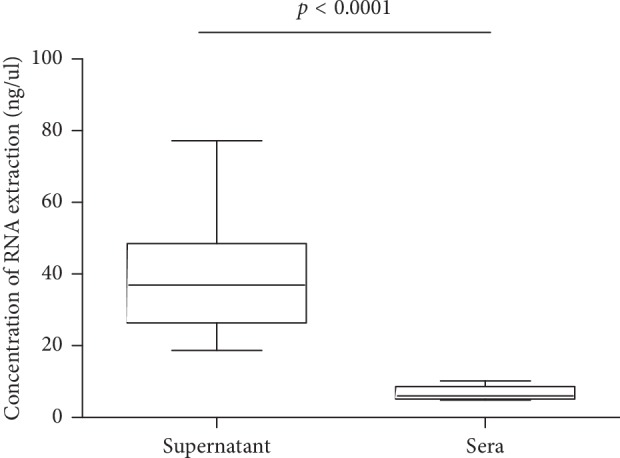
Concentration of cf-RNA extraction (ng/ul) in the MPE supernatant and matched sera as determined by using a spectrometer is plotted on the *y*-axis. Statistically significant differences were determined by using paired Student's *t*-test.

To determine whether delayed MPE supernatant processing would lead to the degradation of cf-RNA, 23 patients with MPE supernatant were collected for cf-RNA assay. The samples of MPE supernatant were placed in 4°C icebox at different time points (0, 24, and 48 hours) and then processed. Results showed that no significant difference was found for the concentrations of cf-RNA at three time points (*p*=0.632; Figure 1). We further detected the *ALK* gene rearrangement by qRT-PCR, nevertheless, there was no difference between the three time points too (Figure 2). We also tested the RNA concentration in the matched sera of patients with MPE (*n* = 23); however, the level of cf-RNA was very low in sera than in the supernatant (*p* < 0.0001; Figure 3).

### 3.3. Comparison of Detection of *ALK* Gene Rearrangement between Sera, Supernatant, and Cell Blocks

**Figure 4:**
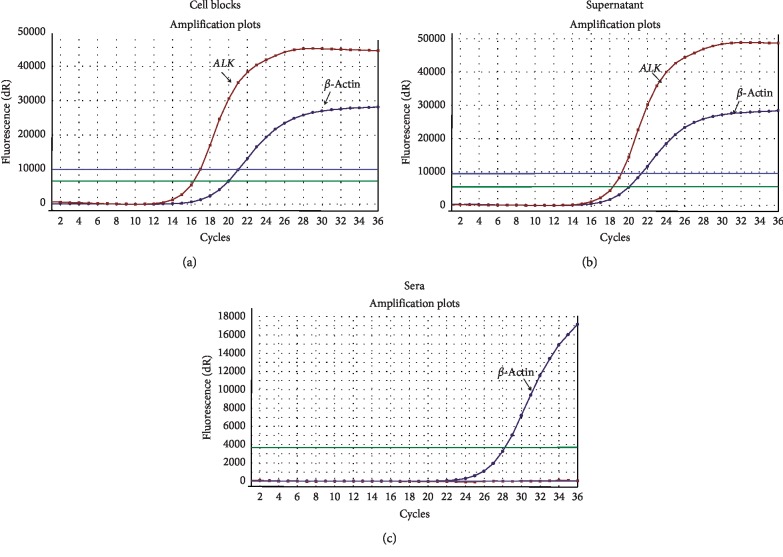
Detection of *ALK* gene rearrangement in cellular RNA of MPE cell blocks and cf-RNA of the MPE supernatant and sera. (a) Amplification curve of *ALK* rearrangement in cellular RNA of MPE cell blocks. (b) Amplification curve of *ALK* rearrangement in cf-RNA of the MPE supernatant. (c) Amplification curve of *ALK* rearrangement in cf-RNA of sera.

Of the 168 samples for which *ALK* gene rearrangement results were available, *ALK*-positive rearrangement of cell blocks was 7.1% (12/168) (Table 1). Cf-RNA of 23 cases of matched sera and supernatant was isolated (including 12 cases of *ALK*-positive rearrangement). Results showed that the detection efficiency of *ALK* gene rearrangement was completely consistent in the supernatant (cf-RNA) and cell blocks (cellular RNA) (McNemar's *p*=1.000; kappa = 1.000; Table 2; Figure 4). However, the detection of *ALK* gene rearrangement was wholly negative in all matched sera (McNemar's *p* < 0.001; kappa = 0.000; Table 2; Figure 4).

## 4. Discussion

MPE is a common manifestation of advanced NSCLC. For many patients, MPE can be used for driver gene status tests, including *EGFR* gene mutation and *ALK* gene rearrangement [4, 6, 7, 13]. Recently studies have showed that the cf-DNA of MPE could be used for the *EGFR* mutation test [10, 11]. However, whether the cf-RNA of MPE is appropriate for *ALK* gene rearrangement testing is not yet known and was therefore the subject of the current study.

We firstly detected the concentration of cf-RNA and *ALK* gene rearrangement in different processing time points (0, 24, and 48 hours), and the results showed that there was no difference between them (Table 1; Figure 2). That indicates that there will be plenty of time to process cf-RNA in clinical working practice for MPE samples. In addition, we found that the concentration of cf-RNA was very low in matched sera than in the supernatant (Figure 3). These findings suggest low level of cf-RNA burden in sera. Previous studies have showed that MPE cell blocks can be used for *ALK* gene rearrangement detection [2, 4]. Hence, in this study, we compared the mutation status in cf-RNA of sera and the supernatant to the mutation status in the cell blocks from the same patients. Our findings showed that the *ALK* status concordance rates were 100% between the supernatant and cell blocks, but 0% between sera and cell blocks in *ALK* gene rearrangement cases (Table 2; Figure 4). We speculated that very low level cf-RNA in sera maybe the cause of negative results, while high concentration of cf-RNA provide enough material to ensure the sensitivity testing *ALK* gene rearrangement status in the supernatant. So, we think that cf-RNA in MPE supernatant samples is one of the proper substrates for *ALK* gene rearrangement testing. In the future, cf-RNA and cf-DNA in the MPE supernatant would be an attractive alternative source supplying useful information about the mutation status of *ALK*, *EGFR*, and other genes.

In conclusion, this study explored a newly developed method for the detection of *ALK* gene rearrangement in MPE. Cf-RNA in the supernatant would be one potential substitute for molecular diagnostics.

## Figures and Tables

**Table 1:** Clinicopathological features of patients with MPE (*n* = 168).

Clinicopathological features	*ALK* positive rearrangement	*ALK* negative rearrangement	*p* value
Age (years)			
<60	10	71	*p*=0.012
≥60	2	85	

Sex			
Male	4	83	*p*=0.184
Female	8	73	

Smoking			
Never	9	79	*p*=0.104
Ever	3	77	

*ALK*, anaplastic lymphoma kinase; chi-square test.

**Table 2:** Comparison of *ALK* gene rearrangement results in supernatant, sera, and cell blocks.

Cell blocks	Supernatant		Sera	
	+	−	+	−
+	12	0	0	12
−	0	11	0	11
*p* value (McNemar's test)	=1.000		≤0.001	
Kappa value	=1.000		=0.000	

+, *ALK* positive rearrangement; −, *ALK* negative rearrangement.

## Data Availability

The data used to support the findings of this study are available from the corresponding author upon request.
